# Characterization of ovarian clear cell carcinoma using target drug-based molecular biomarkers: implications for personalized cancer therapy

**DOI:** 10.1186/s13048-017-0304-9

**Published:** 2017-02-10

**Authors:** Mengjiao Li, Haoran Li, Fei Liu, Rui Bi, Xiaoyu Tu, Lihua Chen, Shuang Ye, Xi Cheng

**Affiliations:** 1 Department of Gynecologic Oncology, Fudan University Shanghai Cancer Center, 270 Dong-an Road, Shanghai, 200032 China; 2 Department of Oncology, Shanghai Medical College, Fudan University, Shanghai, 200032 China; 3 Department of Pathology, Fudan University Shanghai Cancer Center, Shanghai, 200032 China

**Keywords:** Ovarian clear cell carcinoma, Immunohistochemistry, Chemoresistance, Aurora kinase A, Programmed death ligand-1

## Abstract

**Background:**

It has long been appreciated that different subtypes (serous, clear cell, endometrioid and mucinous) of epithelial ovarian carcinoma (EOC) have distinct pathogenetic pathways. However, clinical management, especially chemotherapeutic regimens, for EOC patients is not subtype specific. Ovarian clear cell carcinoma (CCC) is a rare histological subtype of EOC, which exhibits high rates of recurrence and low chemosensitivity. We assessed potential therapeutic targets for ovarian CCC patients through analyzing the variation of drug-based molecular biomarkers expression between ovarian CCC and high-grade serous carcinoma (HGSC).

**Methods:**

Seven candidate drug-based molecular biomarkers, human epidermal growth factor receptor (EGFR), human epidermal growth factor receptor-2 (HER2), phosphatase and tensin homolog deleted on chromosome ten (PTEN), aurora kinase A (AURKA), breast cancer susceptibility gene 1 (BRCA1), breast cancer susceptibility gene 2 (BRCA2) and programmed death-ligand 1 (PD-L1) were measured in 96 ovarian CCC and 113 HGSC by immunohistochemistry in paraffin embedded tissues. The relationship between these biomarkers and clinicopathological factors were explored.

**Results:**

The expression level of four of the seven drug-based molecular biomarkers was markedly different between HGSC and CCC. High expression levels of HER2 and PD-L1 were more commonly observed in CCC patients (12.6% vs 2.7%, 21.1% vs 11.6%, *P* = 0.006, 0.064, respectively), while loss of BRCA1 and BRCA2 expression were more frequently occurred in HGSC patients (72.6% vs 54.3%, 89.4% vs 79.8%, *P* = 0.007, 0.054, respectively). Survival analysis showed that five of seven biomarkers had prognostic values but varied between subtypes. Furthermore, EGFR expressed frequently in CCC patients with endometriosis than in HGSC patients (44.4% vs 8.3%, *P* = 0.049). AURKA and PD-L1 correlated with the resistance to platinum-based chemotherapy in CCC patients (*P* = 0.043, 0.028, respectively) while no similar results were observed in HGSC patients.

**Conclusion:**

Ovarian CCC showed a markedly different expression map of drug-based molecular biomarkers from HGSC, which suggested a new personalized target therapy in this rare subtype.

**Electronic supplementary material:**

The online version of this article (doi:10.1186/s13048-017-0304-9) contains supplementary material, which is available to authorized users.

## Background

The histological types of epithelial ovarian carcinoma (EOC) have distinct clinical and molecular features with each other [1, 2]. To date, EOC patients are mostly provided with the same therapeutic regimens regardless of the distinct histological characteristics of the primary lesion [3]. However, ovarian clear cell carcinoma (CCC), contributing for 4.8–25% of all ovarian carcinomas [4], is known to be less sensitive to platinum-based front-line chemotherapy and to be associated with a poorer prognosis than the more common serous subtype. Moreover, previous studies have shown that the response rate of various second-line chemotherapeutic regimens for recurrent platinum-resistant ovarian CCC was only 1% [5]. Therefore, a further investigation of the mechanism of the chemoresistance and developing new therapeutic targets are needed for effective clinical management of ovarian CCC.

Although the mechanism of carcinogenesis and chemoresistance of ovarian CCC is still unclear, several genetic changes have been widely investigated. Compared with ovarian high-grade serous carcinomas (HGSC), ovarian CCC are generally negative for p53 mutation and have a lower frequency (6.3%) of breast cancer 1 or 2 (BRCA1/2) mutations [6, 7]. A higher frequency of AT rich interactive domain 1A (ARID1A) and phosphatidylinositol-4, 5-bisphosphate 3-kinase catalytic subunit α (PIK3CA) mutations (36%) were also observed in CCC patients [8, 9].

In addition, protein expression studies of ovarian CCC observed five functional protein activating pathways [10], some of which could be potential therapeutic targets: (PI3K)/AKT/mammalian target of rapamycin (mTOR) pathway, hypoxia-inducible factor 1α (HIF-1α)/vascular endothelial growth factor (VEGF) pathway, hepatocyte nuclear factor 1β (HNF-1β) pathway, interleukin 6 (IL-6)/signal transducer and activator of transcription 3 (STAT3) pathway and MET pathway [11–15]. Recently, Sereni et al. detected 117 drug-based protein expression levels of 72 EOC patients by using reverse-phase protein microarray (RPPA) analysis. They found that 11 out of 117 proteins of ovarian CCC (*n* = 7) showed a distinct signaling network from other subtypes of EOC, such as enhanced activation of phosphorylated epidermal growth factor receptor (EGFR) and phosphorylated human epidermal growth factor receptor-2 (HER2) [16]. However, the sample size of ovarian CCC in the study of Sereni et al. was not large enough due to the rarity of this subtype. In the current study, we simultaneously compared seven drug-based protein expression levels (either in an existing FDA-approved therapy or in an experimental therapeutic in clinical trial) both in ovarian CCC and HGSC on a larger scale and investigated their associations with clinical characteristics and survival outcomes.

## Methods

### Patient cohort study

This study was approved by the Institutional Review Board of Fudan University Shanghai Cancer Center (FUSCC). A written informed consent was obtained from all recruited individuals, and each clinical investigation was conducted according to the principles expressed in the Declaration of Helsinki consent. 96 primary ovarian CCC cases and 113 primary HGSC cases from FUSCC were randomly selected between January 2008 and December 2015. Data collection included age at diagnosis, International Federation of Gynecology and Obstetrics (FIGO) stage, residual disease (optimal debulking <1 cm), ascites, endometriosis and chemotherapeutic regimens. Primary patients with early stage (I and II) underwent complete staging surgery and patients with late stage (III and IV) received cytoreductive surgery. The majority of the patients received platinum-based chemotherapy regimens after primary surgery, and patients were followed up every 3 months for the first year, every 6 months for the next 4 years, and annually for the following years thereafter. Progression-free survival (PFS) and overall survival (OS) duration were calculated from the date of first surgery to the date of disease recurrence and death or the last follow-up visit, respectively.

### Tissue microarray (TMA) and Immunohistochemistry (IHC)

The portions of tumor tissue to be used for the tissue microarray were selected from a representative tumor area in the corresponding H&E stained section of each block by two gynecologic pathologists (R B and XY T). A 10 ×12 (120 cores) array was made by the Tissue Bank of FUSCC. Each case has two cores made from separate sources. IHC was performed on 5-μm-thick TMA sections from formalin-fixed, paraffin-embedded tissues. Immunohistochemical staining of EGFR, HER2/neu, PTEN and PD-L1 were run through an automated protocol including heat antigen retrieval (Ventana System). AURKA, BRCA1 and BRCA2 were performed as the following. Briefly, xylene dewaxed and alcohol-rehydrated TMA slides were treated in 10 mM citrate buffer (PH = 6.0) at 100 °C for 10 min, according to the antigen retrieval method. After cooling for 2 h, endogenous peroxidase activity was blocked with 3% hydrogen peroxide solution. Then nonspecific binding was inhibited with nonimmune goat serum (Maixin Biological Technology Development, Fuzhou, China) for 30 min. Antibodies against molecular biomarkers, BRCA1, BRCA2 and AURKA (see Additional file 1: Table S1) were incubated on the tumor tissues for 14 h at 4 °C in a moist chamber, which were followed by a secondary incubation with pre-diluted anti-rabbit HRP for 1 h (Maixin Biological Technology Development, Fuzhou, China). Molecular biomarker signals were visualized after addition of 3, 3’-diaminobenzidine tetrahydrochloride (DAB) for the dilution of 1:50. TMA slides counterstaining was performed with hematoxylin. The positive controls are known positive case samples. And nonimmune goat serum takes the place of primary antibody serving as the negative control.

### TMA scoring

The IHC staining results were scored independently by two gynecologic pathologists (R B and XY T), who were blinded to clinical information of the patients. Scoring system is on the basis of both percentage of positive tumor cells and staining intensity. Staining intensity was graded 0 (none), 1 (weak), 2 (intermediate) or 3 (strong), and distribution of the cellular staining was graded as 0 (none),1 (<10% of the cells), 2 (11%–50% of the cells), 3 (51%–75% of the cells) or 4 (>75% of the cells). The multiplication of intensity and distribution was scored 0 (**≦**3), 1+ (>3 to 6), 2+ (>6 to 9), or 3+ (>9 to 12). Finally, the assessment of the protein expression was defined as negative (<2+) and positive (≧2+ to 3+). Both of cytoplasmic and nuclear positive cores of AURKA were defined as positive. For cores that were uninterpretable caused by tissue loss or lacking of tumor cells, a score of not applicable (N/A) was assigned.

### Statistical analysis

SPSS 19.0 software (IBM, Armonk, New York, USA) was used for the analysis. We performed Kaplan–Meier Model and multivariate Cox proportional hazards regression analysis for survival estimate. Chi-squared test was used to detect the association of molecular biomarker expression level with clinicopathological factors. Each reported *P* value was two sided, and *P* < 0.05 was used to infer statistical significance.

## Results

### Study of the patient cohort

Clinicopathological characteristics of the ovarian HGSC and CCC patients enrolled in the study were summarized in Tables 1 and 2. The median follow-up time was 45.0 months (range 2.0–90.0) in HGSC and 18.0 months (range 2.0–91.0) in CCC. There were 84/108 (77.8%, median time 47.0 months) recurrences, 61/113 (54.0%) deaths in HGSC and 39/91 (42.9%, median time 12.5 months) recurrences, 26/96 (27.1%) deaths in CCC. By using univariate and multivariate Cox proportional hazards regression model, we evaluated potentially prognostic factors in this two subtypes of patients. The results revealed that a shorter overall survival time was associated with late FIGO stage (III + IV) (adjusted HR 3.241, 95% CI 1.065–10.879, *P* = 0.047 in HGSC and HR 4.660 95% CI 1.533–14.167, *P* = 0.007 in CCC, Tables 1 and 2) and chemoresistance (adjusted HR 3.573, 95% CI 2.056–6.210, *P* = 0.000) in HGSC and HR 7.230, 95% CI 1.992–26.237, *P* = 0.003 in CCC, Tables 1 and 2). Besides, another two factors in univariate analysis, residual tumor size >1 cm (*P* =0.042) and ascites (*P* = 0.005), also implied poor prognosis of HGSC and CCC patients, respectively.

**Table 1:** Clinical prognostic features of HGSC patients

Prognostic factors	Cases (%)	Univariate P^a^	Multivariate	
			HR (95% CI)	P^b^
Age (years)		0.281		0.495
≤ 57 (median)	57(50.4)		1.000	
> 57 (median)	56(49.6)		1.010(0.982–1.037)	
FIGO Stage		**0.002**		**0.047**
Early(I + II)	17(15.0)		1.000	
Late(III + IV)	96(85.0)		3.241(1.065–10.879)	
Residual tumor (cm)		**0.042**		0.543
≤ 1	104(92.0)		1.000	
> 1	9(8.0)		1.300 (0.558–3.027)	
Ascites		0.085		0.690
No	11(9.7)		1.000	
Yes	102(90.3)		1.280(0.380–4.311)	
Endometriosis		0.140		0.568
No	101(89.4)		1.000	
Yes	12(10.6)		1.245(0.586–2.647)	
Chemotherapeutic response		**0.000**		**0.000**
Platinum sensitive	77(72.0)		1.000	
Platinum resistant	30(28.0)		3.573(2.056–6.210)	

Log rank test

^a^without adjustment

Cox proportional hazards regression analysis

^b^with adjustment for age, FIGO stage, residual tumor, ascites, endometriosis, and chemotherapeutic response

Bold value denotes *P* with statistical significance

**Table 2:** Clinical prognostic features of CCC patients

Prognostic factors	Cases (%)	Univariate	Multivariate	
	96(100)	P^c^	HR (95% CI)	P^d^
Age (years)		0.524		0.292
≤ 54 (median)	50(52.1)		1.000	
> 54 (median)	46(47.9)		0.980(0.944–1.017)	
FIGO Stage		**0.000**		**0.007**
Early(I + II)	64(66.7)		1.000	
Late(III + IV)	32(33.3)		4.660(1.533–14.167)	
Residual tumor (cm)		0.115		0.541
≤ 1	93(96.9)		1.000	
> 1	3(3.1)		1.690 (0.314–9.080)	
Ascites		**0.005**		0.338
No	53(55.2)		1.000	
Yes	43(44.8)		1.613(0.606–4.290)	
Endometriosis		0.121		0.568
No	78(81.3)		1.000	
Yes	18(18.7)		0.647(0.144–2.894)	
Chemotherapeutic response		**0.000**		**0.003**
Platinum sensitive	64(72.7)		1.000	
Platinum resistant	24(27.3)		7.230(1.992–26.237)	

Log rank test

^c^without adjustment

Cox proportional hazards regression analysis

^d^with adjustment for age, FIGO stage, residual tumor, ascites, endometriosis, and chemotherapeutic response

Bold value denotes *P* with statistical significance

### Ovarian CCC patients showed different expression map of drug-based molecular biomarkers from HGSC patients

**Fig. 1:**
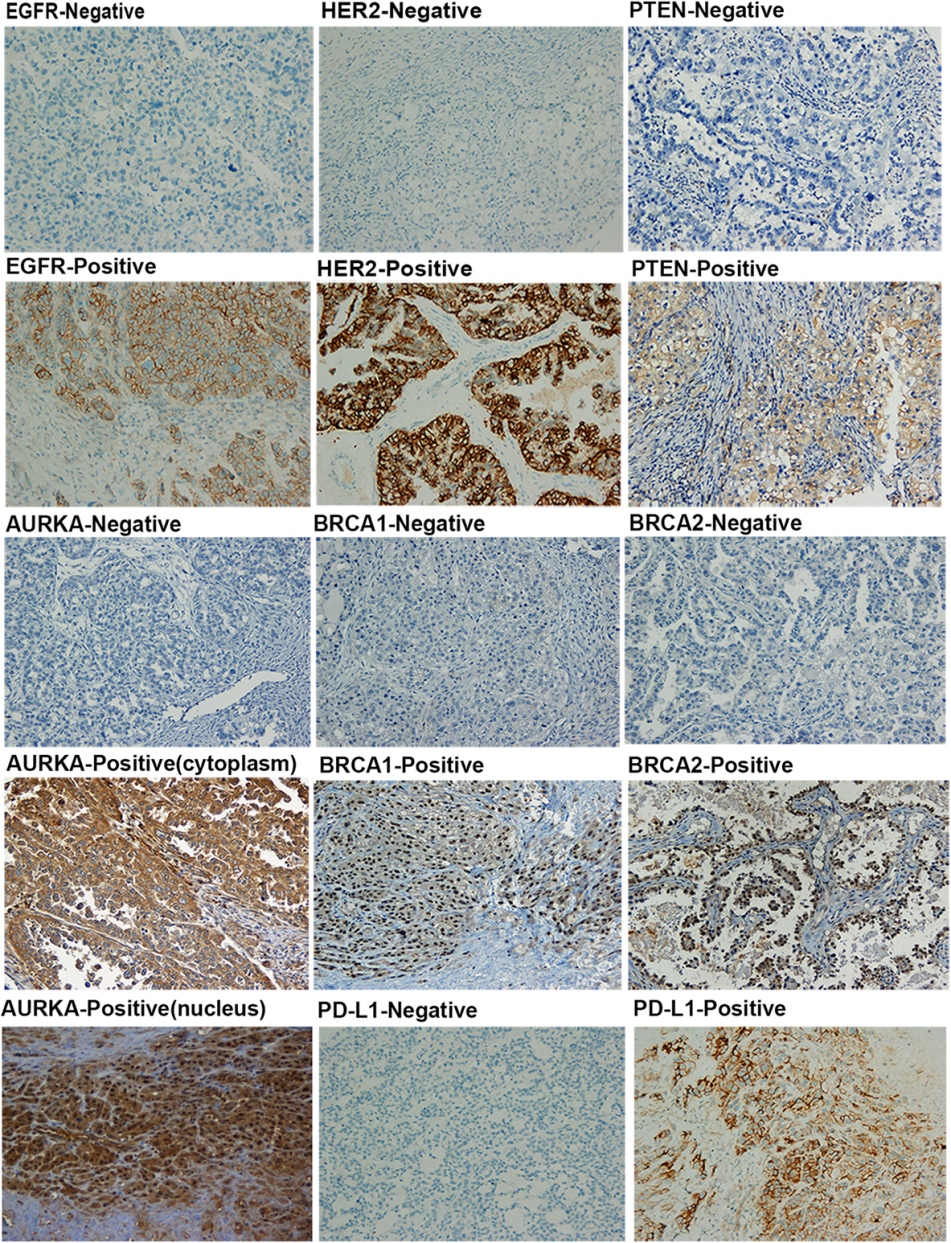
Representative immunostains of seven biomarkers in ovarian clear cell carcinoma (x 200)

**Fig. 2:**
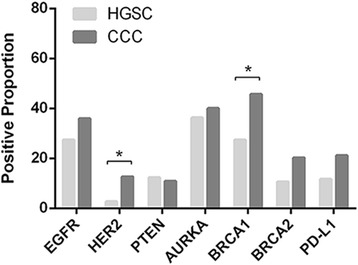
Positive proportion of seven biomarkers in HGSC and CCC patients. CCC patients expressed higher level of HER2 and BRCA1 than HGSC patients (12.6% vs 2.7%, 45.7% vs 27.4%, *P* = 0.006, 0.007 respectively, Chi-squared test)

**Fig. 3:**
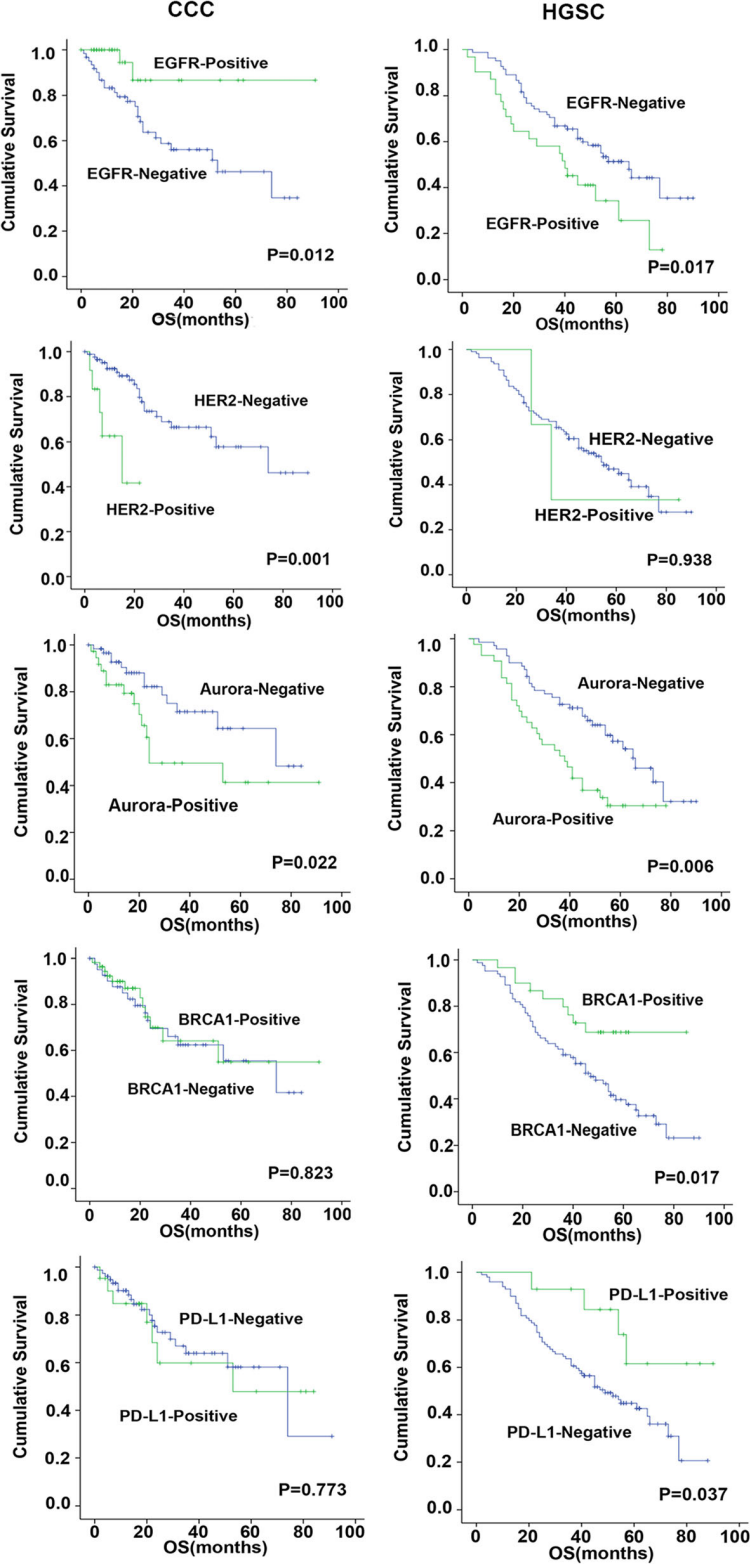
Kaplan–Meier survival curves based on immunohistochemistry results. Five of seven biomarkers (EGFR, HER2, AURKA, BRCA1, PD-L1) are significantly associated with overall survival (OS) in HGSC and/or CCC patients

Typical immunohistochemistry results of the drug-based molecular biomarkers were partly presented in Fig. 1. The results showed a significant difference of biomarker expression map between HGSC and CCC. High levels of HER2 and PD-L1 were commonly observed in CCC patients, compared with HGSC (12.6% vs 2.7%, 21.1% vs 11.6%, respectively, *P* = 0.006, 0.064 respectively, Chi-squared test). Loss of BRCA1 and BRCA2 expression were more frequently occurred in HGSC (72.6% vs 54.3%, 89.4% vs 79.8%, *P* = 0.007, 0.054, respectively). In addition, it showed little difference in positive rates of EGFR, PTEN, and AURKA between the two subtypes (Fig. 2). We further explored the association of the expression level of these markers with PFS/OS in both of the HGSC and CCC patients (Table 3). According to Kaplan-Meier model, five of seven biomarkers showed prognostic value in at least one subtype (Fig. 3). For HGSC patients, we found that poor prognosis were often companied by high expression levels of EGFR, AURKA (*P* = 0.017, 0.006, respectively), while better prognosis were associated with overexpression of BRCA1 and PD-L1 (*P* = 0.017, 0.037, respectively). Interestingly, the results of CCC group exhibited a different biomarker expression map—high expression level of EGFR had a relationship with longer survival time (*P* = 0.012) and overexpression of HER2 and AURKA predicted shorter survival time and progression-free time (*P* =0.001, 0.022, respectively). With multivariate Cox proportional hazards regression analysis, we found that CCC patients with late FIGO stage, low expression of EGFR and high expression of HER2 exhibited shorter survival time, while HGSC patients with high expression of AURKA and low expression of BRCA1 had poorer prognosis (Table 4). Chemoresistance is a strong negative prognostic factor in both of the two subtypes.

**Table 3:** Association of the expression level of molecular biomarkers with PFS/OS

Molecular biomarkers	High-grade serous carcinoma			Clear cell carcinoma			*P* ^e^ value
	Cases (%)	P(OS)	P(PFS)	Cases (%)	P(OS)	P(PFS)	
EGFR	113(100)	**0.017**	**0.042**	92(100)	**0.012**	0.267	0.195
Negative	82(72.6)			59(64.1)			
Positive	31(27.4)			33(35.9)			
HER2	113(100)	0.938	0.475	95(100)	**0.001**	0.200	**0.006**
Negative	110(97.3)			83(87.4)			
Positive	3(2.7)			12(12.6)			
PTEN	113(100)	0.323	0.397	93(100)	0.160	0.061	0.716
Negative	99(87.6)			83(89.2)			
Positive	14(12.4)			10(10.8)			
AURKA	113(100)	**0.006**	0.382	95(100)	**0.022**	**0.035**	0.582
Negative	72(63.7)			57(60.0)			
Positive	41(36.3)			38(40.0)			
BRCA1	113(100)	**0.017**	**0.032**	92(100)	0.823	0.424	**0.007**
Negative	82(72.6)			50(54.3)			
Positive	31(27.4)			42(45.7)			
BRCA2	113(100)	0.743	0.500	94(100)	0.466	0.929	0.054
Negative	101(89.4)			75(79.8)			
Positive	12(10.6)			19(20.2)			
PD-L1	113(100)	**0.037**	0.159	95(100)	0.773	0.425	0.064
Negative	99(88.4)			75(78.9)			
Positive	13(11.6)			20 (21.1)			

A total of 113 cases of HGSC and 96 cases of CCC have both primary tumor immunohistochemistry results and survival information. However, the total number for each antibody does not add to up 96 in CCC group

^a^Chi-squared tests were performed for the difference of positive rates between HGSC and CCC groups

Bold value denotes P with statistical significance

**Table 4:** Multivariate cox analyses of biomarkers and clinical factors with OS

	High-grade serous carcinoma		Clear cell carcinoma	
Factors	Hazard Ratio (95% CI)	HGSC P	Hazard Ratio (95% CI)	CCC P
Stage				
Early(I + II)	1.000		1.000	
Late(III + IV)	3.320(0.965–11.427)	0.057	9.703(2.588–36.381)	**0.001**
Residual tumor				
< 1 cm	1.000		1.000	
> 1 cm	1.367(0.560–3.339)	0.492	1.400(0.244–8.032)	0.706
Ascites				
No	1.000		1.000	
Yes	0.994(0.293–3.373)	0.993	2.587(0.848–7.890)	0.095
Chemotherapeutic response				
Sensitive	1.000		1.000	
Resistant	3.803(2.170–6.664)	**0.000**	3.562(1.063–11.936)	**0.040**
EGFR				
Negative	1.000		1.000	
Positive	1.244(0.653–2.370)	0.507	0.044(0.003–0628)	**0.021**
HER2				
Negative	1.000		1.000	
Positive	1.277(0.299–5.456)	0.741	7.948(1.745–36.208)	**0.007**
PTEN				
Negative	1.000		1.000	
Positive	0.696(0.257–1.880)	0.474	4.475(0.825–24.274)	0.082
AURKA				
Negative	1.000		1.000	
Positive	2.082(1.162–3.730)	**0.014**	1.763(0.616–5.044)	0.291
BRCA1				
Negative	1.000		1.000	
Positive	0.433(0.211–0.885)	**0.022**	1.934(0.653–5.738)	0.235
BRCA2				
Negative	1.000		1.000	
Positive	1.671(0.755–3.701)	0.206	1.258(0.352–4.493)	0.723
PD-L1				
Negative	1.000		1.000	
Positive	0.520(0.182–1.487)	0.222	1.157(0.387–3.456)	0.794

*CI* confidence interval

Bold value denotes P with statistical significance

### Expression of drug-based molecular biomarkers in ovarian CCC exhibited various relationships to clinicopathological factors

**Fig. 4:**
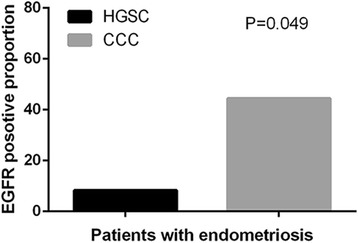
Overexpression of EGFR was more frequently observed in CCC patients with endometriosis than in HGSC patients (44.4% vs 8.3%, *P* = 0.049, Chi-squared test)

We further analyzed the relationship between biomarkers expression level and clinicopathological factors (Additional file 1: Table S2A and B). The results showed that HGSC patients with late stage were more likely to lose PTEN expression (*P* = 0.036), while CCC patients have a higher rate of PTEN silence in early stage instead of late stage (*P* = 0.283). Since endometriosis plays a crucial part in the carcinogenesis of CCC, we investigated potential relationship between these biomarkers and endometriosis. Although no significant results were observed, CCC patients with endometriosis showed a higher expression rate of EGFR compared with HGSC patients (Fig. 4, 44.4% vs 8.3%, *P* = 0.049). To explore candidate chemotherapeutic targets, we verified associations of these biomarkers with chemotherapeutic response as well as recurrence status (Chi-squared test, Table 5 and Additional file 1: Table S3). When the two different histopathological subgroups were compared, it was noted that positive AURKA and PD-L1 staining were observed more frequently in CCC patients with platinum-based chemotherapeutic resistance (*P* = 0.042, 0.028, respectively). In addition, as discussed earlier, another striking finding was that EGFR showed a markedly lower expression level in recurrent CCC patients, compared with no recurrent CCC patients (*P* = 0.023). Interestingly, no similar phenomenon was observed in HGSC patients. Then we stratified recurrent cases based on the response to platinum-based chemotherapy, and no significant association was observed (Additional file 1: Table S4).

**Table 5:** Correlation of molecular biomarker expression and chemotherapeutic response

Molecular biomarkers	HGSC			CCC		
	Platinum sensitive (%)	^a^Platinum resistant (%)	HGSCP	Platinum sensitive (%)	^a^Platinum resistant (%)	CCC P
EGFR			0.365			0.129
Negative	58(75.3)	20(66.7)		36(58.1)	17(77.3)	
Positive	19(24.7)	10(33.3)		26(41.9)	5(22.7)	
HER2			1.000			0.725
Negative	75(97.4)	29(96.7)		56(87.5)	19(82.6)	
Positive	2(2.6)	1(3.3)		8(12.5)	4(17.4)	
PTEN			0.530			0.679
Negative	68(88.3)	25(83.3)		58(92.1)	21(87.5)	
Positive	9(11.7)	5(16.7)		5(7.9)	3(12.5)	
AURKA			0.505			**0.042**
Negative	49(63.6)	17(56.7)		42(65.6)	10(41.7)	
Positive	28(36.3)	13(43.3)		22(34.4)	14(58.3)	
BRCA1			0.584			0.848
Negative	55(71.4)	23(76.7)		35(56.5)	13(54.2)	
Positive	22(28.6)	7(23.3)		27(43.5)	11(45.8)	
BRCA2			1.000			0.542
Negative	68(88.3)	27(90.0)		50(80.6)	21(87.5)	
Positive	9(11.7)	3(10.0)		12(19.4)	3(12.5)	
PD-L1			0.502			**0.028**
Negative	66(86.8)	28(93.3)		56(87.5)	15(65.2)	
Positive	10(13.2)	2(6.7)		8(12.5)	8(34.8)	

There are 107 cases of HGSC and 88 cases of CCC having both primary tumor immunohistochemistry results and information of chemotherapy. However, the total number for each antibody does not add to up 107 and 88, respectively

^a^Interval time <6 months from completion of last platinum-based chemotherapy to disease recurrence

Bold value denotes P with statistical significance

## Discussion

Deciphering ovarian CCC molecular expression patterns is important not only for understanding the pathogenesis of this rare disease but also for exploring therapeutic options for patients who are chemotherapeutic resistant. In this study, we investigated the molecular characterization of CCC patients compared with HGSC patients on several actionable drug-based molecular biomarkers. These molecular biomarkers might be valuable for predicting the outcome and the response to chemotherapeutic regimens for CCC, compared with the classical clinicopathological prognostic factors [17].

EGFR and HER2 are the members of the HER family, whose EGF signaling pathway is known to play a crucial role in tumor initiation, progression and metastasis. In this study, EGFR and HER2 protein were more frequently expressed in CCC patients than in HGSC patients (35.9% vs 27.4%, 12.6% vs 2.7%, respectively). Besides, Aikou Okamoto et al. observed a gene amplification of EGFR in CCCs and it was related with endometriosis [18]. In line with this, our finding showed that there was 44.4% CCC patients with endometriosis expressed high level of EGFR, while only 8.3% patients with endometriosis in HGSC have high expression of EGFR (Fig. 4, *P* = 0.049). Tan et al. also reported that HER2 gene amplification and protein overexpression were observed in 14% of CCCs through hierarchical cluster analysis [19]. However, Friedlander ML et al. demonstrated that only 2.6% of pure CCCs expressed high level of HER2 protein by IHC tests, but 9.4% of CCCs showed HER2 gene amplification by fluorescent in situ hybridization (FISH) tests [20]. These inconsistent results might be caused by differences in the scale of cohorts and criteria of the cut-off value for positive cases. Moreover, evidence on the prognostic value of EGFR and HER2 with respect to survival is still inconclusive in ovarian cancer [21]. We observed a favorable prognostic value of EGFR in CCCs and a reverse prognostic value in HGSCs. This contrasting result could be explained by the phenomenon that the positive rate of EGFR of CCC patients in early stage (I + II) was much higher than of HGSC patients in early stage (39.7% vs 17.7%, Additional file 1: Table S2A and B), which implied that EGFR might play a favorable part in the earlier progression of CCC. And previous studies either focused on stage III/IV ovarian patients, or performed IHC assay in a smaller sample size of CCC because of the rarity of this subtype, which might cause bias. In addition, Wang et al. showed that high expression of EGFR in tumor stroma, rather than in tumor cells, correlates with aggressive clinical features in epithelial ovarian cancer, and is an independent prognostic factor [22]. This complexity of the role of EGF signaling pathway in ovarian cancer explained, at least in part, why several clinical trials performed on patients with positive EGFR or HER2 immunostaining or genetic changes did not show satisfying response to HER-targeted therapy [23–25].

TCGA (The Cancer Genome Atlas) analyses showed that the BRCA1 and BRCA2 mutations in 22% of the high-grade serous ovarian cancer samples triggered a wide range of aberrations in DNA damage repair pathways, such as homologous repair pathway [26]. Patients with germline BRCA mutation gained great clinical benefit from using PARP (Poly ADP-ribose polymerase) inhibitors, olaparib for instance, in clinical trials [27]. Beyond this, Konstantinopoulos et al. found that BRCAness profile (patients with sporadic cancers that have a BRCAness phenotype, characterized by defective homologous repair) correlates with responsiveness to platinum and PARP inhibitors and identifies a subset of patients with improved outcome [28–30]. Our analysis revealed that loss of BRCA1 expression was more frequently occurred in HGSC patients than in CCC patients, predicting a shorter overall survival time (*P* = 0.017), which implied potential benefits of PARP inhibitors. However, it is suggested a deeper exploration of the relationship between genetic changes and protein expression of BRCA1/2 in sporadic ovarian cancer. In addition to somatic BRCA1/2 mutation, PTEN loss may be a common contributing event causing homologous repair dysfunction. Previous studies have shown that ovarian CCC often exhibit genetic alterations of PTEN in 5% CCC patients [31], and preclinical researched showed that ovarian cell lines with PTEN mutation were more sensitive to PARP inhibitors [32]. In the present study, we observed a much higher level of PTEN loss in both of HGSCs (87.6%) and CCCs (89.2%), compared with previous studies [17, 20]. This might be due to the different efficacy of the antibodies used in IHC test. According to our analyses (Additional file 1: Table S2A and B), CCCs have a higher rate of PTEN silence in early stage than HGSCs (92.0% vs 70.5%, respectively). However, targeting PTEN only is not enough due to tumors with ARID1A mutation also frequently harbor PTEN or PIK3CA mutation in CCC [33, 34]. Therefore, it is worth trying to use PARP inhibitors for patients with sporadic cancers who lose BRCA or PTEN expression, as well as in combination with other target drugs if necessary.

Aurora kinase A (AURKA) is involved with centrosome function, mitotic entry and spindle assembly in cells [35]. Recently AURKA has been extensively investigated in different neoplasms, including breast, ovarian, colon, liver, pancreas, bladder, and gastric carcinomas [36]. Even if the molecular mechanism of AURKA is complex [37, 38], several researches implied that AURKA protein expression is strongly linked with poor patient outcome and aggressive disease characteristics of ovarian cancer [39, 40]. Consistently, we found the same effects of AURKA in CCC patients that both of the overall survival time and progression-free time were shortened in CCCs with high expression level of AURKA (*P* < 0.05, Table 3). In addition, overexpression of AURKA also had a relationship with platinum-based chemotherapeutic resistance in CCC patients, suggesting that targeted at AURKA might provide a promising therapeutic option. Most recently, ENMD-2076, acting by blocking AURKA and tyrosine kinase enzymes from working and stopping new blood vessels from growing, is in phase II clinical trial designed specifically for CCC patients (ClinicalTrials.gov number, NCT01914510). The result will be posted in 2018.

Surgery and chemotherapy is the mainstay of ovarian cancer treatment. However, with the development of immunology, immunotherapy represents a rational and alternative approach for ovarian patients, especially for those who have recurrent and/or chemotherapeutic resistant diseases [41–43]. Programmed death 1 (PD-1) and its ligand PD-L1 play a crucial role in the inhibition of T cell-mediated immune response. Binding of PD-L1 to PD-1 causes the exhaustion of effector T cells and immune escape of tumor cells, leading to poor prognostic outcome. Previous studies have shown that overexpression of PD-L1 in cancers such as gastric cancer, hepatocellular carcinoma, renal cell carcinoma and esophageal cancer, pancreatic cancer and bladder cancer is related to poor prognosis. However, PD-L1 expression correlates with better clinical outcomes in breast cancer and Merkel cell carcinoma [44]. The prognostic value of PD-L1 in ovarian cancer is controversial [45, 46]. A recent study of 490 EOC cases revealed that PD-L1 had a positive association with survival in high-grade serous subtype rather than clear cell subtype [45]. Accordingly, our results also showed that PD-L1 expression was correlated with a favorable prognosis of HGSC patients (Fig. 2, *P* = 0.037), which might be caused by an immunological stalemate that activation of T cells triggered a negative feedback in the microenvironment around tumor cells. Another study reported by Friedlander ML et al. showed that only 7.4% of CCC cases have aberrant PD-L1 overexpression through IHC method. However, the sample size in Friedlander’s study is too small (*n* = 27) and no detailed clinical information to further analyze [20]. By performing analysis on a larger scale (*n* = 96), we found that there was no significant correlation between PD-L1 expression and prognosis in CCC patients, but higher expression of PD-L1 was observed in this subtype than in HGSC (21.1% vs 11.6%, respectively). And by combining with the analysis of chemotherapeutic response, it is revealed that CCC patients with high expression level of PD-L1 tend to be more resistant to platinum-based chemotherapy (*P* = 0.028), which suggested that PD-L1 might play a part in the chemoresistance of CCC patients.

It is true that different studies may probably draw different conclusions from TMA immunohistochemistry (IHC) because of various reasons, such as the efficacy of antibodies, intra-tumor heterogeneity, criteria of the cut-off value for positive cores and sufficient follow-up time of patients. Although we have tried our best to balance these factors, there are still some limitations in our study. Firstly, since there being no uniform interpretations for IHC scoring and analyses, we used the same cut-off value for all antibodies, which might not be optimal for each marker. Secondly, we did not perform correction for multiple comparisons in some of our analyses. While IHC assay could only detect protein expression changes, DNA sequencing is still needed to further investigate genes status for these molecular biomarkers. Indeed, our work is exploratory and descriptive, we would try to solve these problems and perform larger cohort studies combined with genomic changes in the next future.

## Conclusions

Ovarian clear cell carcinoma showed a markedly different biomarker protein expression map from high-grade serous carcinoma. Expression level of HER2 and PD-L1 were higher in CCC patients, compared with HGSC. Besides, overexpression of AURKA and PD-L1 significantly associated with chemoresistance in CCC. All these differences suggested newly designed clinical trials of candidate target drugs for this rare subtype, and deserved further investigation of potential molecular mechanisms.

## Additional file

Additional file 1: Table S1. Information of antibodies used in immunohistochemistry. **Table S2A.** Relationship with clinicopathological factors-HGSC. **Table S2B.** Relationship with clinicopathological factors-CCC. **Table S3** Association molecular biomarkers expression and platinum-based chemotherapeutic response. **Table S4.** Comparison of molecular biomarkers between recurrent and disease-free patients. (DOCX 42 kb)

## Abbreviations

AURKA: Aurora kinase A
BRCA1: Breast cancer susceptibility gene 1
BRCA2: Breast cancer susceptibility gene 2
CCC: Clear cell carcinoma
CI: Confidence interval
EGFR: Epidermal growth factor receptor
EOC: Epithelial ovarian carcinoma
FIGO: International Federation of Gynecology and Obstetrics
HER2: Human epidermal growth factor receptor 2
HGSC: High-grade serous carcinoma
HR: Hazard ratio
OS: Overall survival
PD-L1: Programmed death-ligand 1
PFS: Progression-free survival
PTEN: Phosphatase and tensin homolog deleted on chromosome ten
