# Supplementary material for: Characterization of ovarian clear cell carcinoma using target drug-based molecular biomarkers: implications for personalized cancer therapy
Source: J Ovarian Res. 2017 Feb 10;10:9. doi: 10.1186/s13048-017-0304-9 (PMC5303290; doi:10.1186/s13048-017-0304-9)
Supplement: Additional file 1: Table S1. — Information of antibodies used in immunohistochemistry. Table S2A. Relationship with clinicopathological factors-HGSC. Table S2B. Relationship with clinicopathological factors-CCC. Table S3 Association molecular biomarkers expression and platinum-based chemotherapeutic response. Table S4. Comparison of molecular biomarkers between recurrent and disease-free patients. (DOCX 42 kb) [file 13048_2017_304_MOESM1_ESM.docx]

**Additional file 1**

**Table S1** Information of antibodies used in immunohistochemistry

| Name | Supplier | Cat# | Host | Clone | Dilution |
| --- | --- | --- | --- | --- | --- |
| EGFR | Ventana | 790-4347 | Rabbit | 5B7 | Ventana system |
| HER2/neu | Ventana | 790-4493 | Rabbit | 4B5 | Ventana system |
| PTEN | Gene Tech | GT201029 | Mouse | 17.A | Ventana system |
| AURKA | Proteintech | 10297-1-AP | Rabbit | polyclone | 1:300 |
| BRCA1 | Proteintech | 20649-1-AP | Rabbit | polyclone | 1:300 |
| BRCA2 | Proteintech | 19791-1-AP | Rabbit | polyclone | 1:300 |
| PD-L1 | Ventana | 790-4905 | Rabbit | SP263 | Ventana system |

| **IHC Scores of Molecular Biomarkers** |  | **Stage** | | **Residual tumor** | | **Ascites** | | **Endometriosis** | |
| --- | --- | --- | --- | --- | --- | --- | --- | --- | --- |
|  | **Total** | **early** | **late** | **<1cm** | **>1cm** | **no** | **yes** | **no** | **yes** |
| **EGFR** | 113(100) | P^a^ =0.393 |  | P=0.255 |  | P=0.724 |  | P=0.174 |  |
| **0** | 71(62.8) | 11(64.7) | 60(62.5) | 67(64.4) | 4(44.4) | 7(63.6) | 64(62.7) | 60(59.4) | 11(91.7) |
| **1+** | 11(9.7) | 3(17.6) | 8(8.3) | 10(9.6) | 1(11.2) | 2(18.2) | 9(8.8) | 11(10.9) | 0(0) |
| **2+** | 27(23.9) | 2(11.8) | 25(26.0) | 23(22.1) | 4(44.4) | 2(18.2) | 25(24.5) | 26(25.7) | 1(8.3) |
| **3+** | 4(3.6) | 1(5.9) | 3(3.2) | 4(3.9) | 0(0) | 0(0) | 4(4.0) | 4(4.0) | 0(0) |
| **HER2** | 113(100) | P=1.000 |  | P=1.000 |  | P=1.000 |  | P=1.000 |  |
| **0** | 96(85.0) | 16(94.1) | 80(83.3) | 88(84.6) | 8(88.8) | 10(90.9) | 86(84.3) | 85(84.2) | 11(91.7) |
| **1+** | 14(12.3) | 1(5.9) | 13(13.5) | 13(12.5) | 1(11.2) | 1(9.1) | 13(12.7) | 13(12.9) | 1(8.3) |
| **2+** | 3(2.7) | 0(0) | 3(3.2) | 3(2.9) | 0(0) | 0(0) | 3(2.9) | 3(2.9) | 0(0) |
| **3+** | 0(0) | 0(0) | 0(0) | 0(0) | 0(0) | 0(0) | 0(0) | 0(0) | 0(0) |
| **PTEN** | 113(100) | P=**0.036** |  | P=0.309 |  | P=1.000 |  | P=0.169 |  |
| **0** | 87(77.0) | 10(58.8) | 77(80.2) | 80(76.9) | 7(77.8) | 6(54.5) | 81(79.4) | 79(78.2) | 8(66.7) |
| **1+** | 12(10.6) | 2(11.8) | 10(10.4) | 12(11.5) | 0(0) | 4(36.4) | 8(7.8) | 11(10.9) | 1(8.3) |
| **2+** | 12(10.6) | 5(29.4) | 7(7.3) | 11(10.6) | 1(11.1) | 1(9.1) | 11(10.8) | 11(10.9) | 1(8.3) |
| **3+** | 2(1.8) | 0(0) | 2(2.1) | 1(1.0) | 1(11.1) | 0(0) | 2(2.0) | 0(0) | 2(16.7) |
| **AURKA** | 113(100) | P=0.927 |  | P=0.721 |  | P=0.323 |  | P=0.348 |  |
| **0** | 30(26.5) | 6(35.3) | 24(25.0) | 28(26.9) | 2(22.2) | 3(27.3) | 27(25.9) | 28(27.7) | 2(16.7) |
| **1+** | 42(37.2) | 5(29.4) | 37(38.5) | 39(37.5) | 3(33.3) | 6(54.5) | 36(34.6) | 38(37.6) | 4(33.3) |
| **2+** | 41(36.3) | 6(35.3) | 35(36.5) | 37(35.6) | 4(44.5) | 2(18.2) | 39(37.5) | 35(34.7) | 6(50.0) |
| **3+** | 0(0) | 0(0) | 0(0) | 0(0) | 0(0) | 0(0) | 0(0) | 0(0) | 0(0) |
| **BRCA1** | 113(100) | P=0.777 |  | P=0.440 |  | P=1.000 |  | P=1.000 |  |
| **0** | 49(43.4) | 10(58.8) | 39(40.6) | 45(43.3) | 4(44.4) | 6(54.5) | 43(41.3) | 44(43.6) | 5(41.7) |
| **1+** | 33(29.2) | 3(17.6) | 30(31.3) | 29(27.9) | 4(44.4) | 2(18.2) | 31(29.8) | 29(28.7) | 4(33.3) |
| **2+** | 20(17.7) | 4(23.6) | 16(16.7) | 19(18.3) | 1(11.2) | 3(27.3) | 17(16.3) | 17(16.8) | 3(25.0) |
| **3+** | 11(9.7) | 0(0) | 11(11.4) | 11(10.5) | 0(0) | 0(0) | 11(10.6) | 11(10.9) | 0(0) |
| **BRCA2** | 113(100) | P=0.081 |  | P=0.594 |  | P=1.000 |  | P=0.357 |  |
| **0** | 91(80.5) | 11(64.7) | 80(83.3) | 84((80.8) | 7(77.8) | 8(72.7) | 83(79.8) | 80(79.2) | 11(91.7) |
| **1+** | 10(8.8) | 2(11.8) | 8(8.3) | 8(7.7) | 2(22.2) | 2(18.2) | 8(7.7) | 9(8.9) | 1(8.3) |
| **2+** | 11(9.7) | 4(23.5) | 7(7.3) | 11(10.6) | 0(0) | 1(9.1) | 10(9.6) | 11(10.9) | 0(0) |
| **3+** | 1(1.0) | 0(0) | 1(1.1) | 1(0.9) | 0(0) | 0(0) | 1(0.9) | 1(1.0) | 0(0) |
| **PD-L1** | 113(100) | P=0.109 |  | P=0.595 |  | P=0.614 |  | P=1.000 |  |
| **0** | 87(77.0) | 11(64.7) | 76(79.2) | 81(77.9) | 6(66.7) | 6(54.5) | 81(77.9) | 77(76.2) | 10(83.4) |
| **1+** | 13(11.5) | 2(11.8) | 11(11.5) | 10(9.6) | 3(33.3) | 3(27.3) | 10(9.6) | 12(11.9) | 1(8.3) |
| **2+** | 13(11.5) | 4(23.5) | 9(9.3) | 13(12.5) | 0(0) | 2(18.2) | 11(10.5) | 12(11.9) | 1(8.3) |
| **3+** | 0(0) | 0(0) | 0(0) | 0(0) | 0(0) | 0(0) | 0(0) | 0(0) | 0(0) |

**Table S2A** Relationship with clinicopathological factors-HGSC

^a^Chi-squared tests for relationship with clinicopathological factors between positive (IHC score: 2~3+) group and negative (IHC score: 0~1+) group

Bold value denotes P with statistical significance.

**Table S2B** Relationship with clinicopathological factors-CCC

| **IHC Scores of Molecular Biomarkers** |  | **Stage** | | **Residual tumor** | | **Ascites** | | **Endometriosis** | |
| --- | --- | --- | --- | --- | --- | --- | --- | --- | --- |
|  | **Total** | **early** | **late** | **<1cm** | **>1cm** | **no** | **yes** | **no** | **yes** |
| **EGFR** | 92(100) | P^b^ =0.261 |  | P=0.550 |  | P=0.367 |  | P=0.398 |  |
| **0** | 51(55.4) | 33(52.4) | 18(62.1) | 49(55.1) | 2(66.7) | 24(48.0) | 27(64.3) | 40(54.1) | 11(61.1) |
| **1+** | 8(8.7) | 5(7.9) | 3(10.3) | 7(7.9) | 1(33.3) | 6(12.0) | 2(4.8) | 8(10.8) | 0(0) |
| **2+** | 17(18.5) | 12(19.0) | 5(17.2) | 17(19.1) | 0(0) | 13(26.0) | 4(9.5) | 14(18.9) | 3(16.7) |
| **3+** | 16(17.4) | 13(20.7) | 3(10.4) | 16(17.9) | 0(0) | 7(14.0) | 9(21.4) | 12(16.2) | 4(21.2) |
| **HER2** | 95(100) | P=0.519 |  | P=1.000 |  | P=0.789 |  | P=1.000 |  |
| **0** | 57(60.0) | 35(54.7) | 22(71.0) | 54(58.7) | 3(100) | 30(57.7) | 27(62.8) | 47(61.0) | 10(55.5) |
| **1+** | 26(27.4) | 22(34.4) | 4(12.9) | 26(28.3) | 0(0) | 15(28.8) | 11(25.6) | 20(26.0) | 6(33.3) |
| **2+** | 3(3.2) | 2(3.1) | 1(3.2) | 3(3.3) | 0(0) | 2(3.8) | 1(2.3) | 3(3.9) | 0(0) |
| **3+** | 9(9.4) | 5(7.8) | 4(12.9) | 9(9.7) | 0(0) | 5(9.7) | 4(9.3) | 7(9.1) | 2(11.2) |
| **PTEN** | 93(100) | P=0.283 |  | P=1.000 |  | P=0.751 |  | P=1.000 |  |
| **0** | 73(78.4) | 51(81.0) | 22(73.3) | 70(77.8) | 3(100) | 40(78.4) | 33(78.6) | 60(79.0) | 13(76.4) |
| **1+** | 10(10.8) | 7(11.1) | 3(10.0) | 10(11.1) | 0(0) | 6(11.8) | 4(9.5) | 8(10.5) | 2(11.8) |
| **2+** | 10(10.8) | 5(7.9) | 5(16.7) | 10(11.1) | 0(0) | 5(9.8) | 5(11.9) | 8(10.5) | 2(11.8) |
| **3+** | 0(0) | 0(0) | 0(0) | 0(0) | 0(0) | 0(0) | 0(0) | 0(0) | 0(0) |
| **AURKA** | 95(100) | P=0.858 |  | P=0.562 |  | P=0.449 |  | P=0.240 |  |
| **0** | 29(30.5) | 19(29.7) | 10(32.3) | 29(31.5) | 0(0) | 17(32.7) | 12(27.9) | 22(28.6) | 7(38.9) |
| **1+** | 28(29.5) | 20(31.3) | 8(25.8) | 27(29.3) | 1(33.3) | 17(32.7) | 11(25.6) | 21(27.3) | 7(38.9) |
| **2+** | 31(32.6) | 20(31.3) | 11(35.5) | 29(31.5) | 2(66.7) | 16(30.8) | 15(34.9) | 28(36.4) | 3(16.7) |
| **3+** | 7(7.3) | 5(7.7) | 2(6.4) | 7(7.7) | 0(0) | 2(3.8) | 5(11.6) | 6(7.7) | 1(5.5) |
| **BRCA1** | 92(100) | P=0.229 |  | P=0.247 |  | P=0.942 |  | P=0.897 |  |
| **0** | 24(26.1) | 14(22.6) | 10(33.3) | 23(25.8) | 1(33.3) | 13(26.0) | 11(26.2) | 18(24.0) | 6(35.3) |
| **1+** | 26(28.3) | 18(29.0) | 8(26.7) | 25(28.1) | 1(33.3) | 14(28.0) | 12(28.6) | 23(30.7) | 3(17.6) |
| **2+** | 12(13.0) | 5(8.1) | 7(23.3) | 11(12.4) | 1(33.4) | 4(8.0) | 8(19.0) | 10(13.3) | 2(11.8) |
| **3+** | 30(32.6) | 25(40.3) | 5(16.7) | 30(33.7) | 0(0) | 19(38.0) | 11(26.2) | 24(32.0) | 6(35.3) |
| **BRCA2** | 94(100) | P=0.489 |  | P=1.000 |  | P=0.057 |  | P=0.513 |  |
| **0** | 60(63.8) | 38(60.3) | 22(71.0) | 58(63.7) | 2(66.7) | 27(52.9) | 33(76.7) | 49(64.5) | 11(61.1) |
| **1+** | 15(16.0) | 11(17.5) | 4(12.9) | 14(15.4) | 1(33.3) | 10(19.6) | 5(11.6) | 13(17.1) | 2(11.1) |
| **2+** | 17(18.1) | 12(19.0) | 5(16.1) | 17(18.7) | 0(0) | 13(25.5) | 4(9.3) | 13(17.1) | 4(22.2) |
| **3+** | 2(2.1) | 2(3.2) | 0(0) | 2(2.2) | 0(0) | 1(2.0) | 1(2.4) | 1(1.3) | 1(5.6) |
| **PD-L1** | 95(100) | P=0.778 |  | P=0.512 |  | P=0.123 |  | P=0.756 |  |
| **0** | 68(71.5) | 44(68.8) | 24(77.4) | 66(71.7) | 2(66.7) | 31(59.6) | 37(86.0) | 54(70.1) | 14(77.8) |
| **1+** | 7(7.4) | 6(9.4) | 1(3.2) | 7(7.6) | 0(0) | 7(13.5) | 0(0) | 6(7.8) | 1(5.6) |
| **2+** | 18(19.0) | 12(18.8) | 6(19.4) | 17(18.5) | 1(33.3) | 12(23.1) | 6(14.0) | 16(20.8) | 2(11.0) |
| **3+** | 2(2.1) | 2(3.0) | 0(0) | 2(2.2) | 0(0) | 2(3.8) | 0(0) | 1(1.3) | 1(5.6) |

^b^Chi-squared tests for relationship with clinicopathological factors between positive (IHC score: 2~3+) group and negative (IHC score: 0~1+) group

Bold value denotes P with statistical significance.

**Table S3** Association molecular biomarkers expression and platinum-based chemotherapeutic response

| **Molecular biomarkers** | **Recurrent HGSC** | | | **Recurrent CCC** | | |
| --- | --- | --- | --- | --- | --- | --- |
|  | **Platinum**  **Sensitive(%)** | **Platinum**  **Resistant(%)** | **HGSC**  **P** | **Platinum**  **Sensitive(%)** | **Platinum**  **Resistant(%)** | **CCC**  **P** |
| **EGFR** |  |  | 0.767 |  |  | 1.000 |
| negative | 37(69.8) | 20(66.7) |  | 9(75.0) | 17(77.2) |  |
| positive | 16(30.2) | 10(33.3) |  | 3(25.0) | 5(22.7) |  |
| **HER2** |  |  | 1.000 |  |  | 0.634 |
| negative | 51(96.2) | 29(96.7) |  | 12(92.3) | 19(82.6) |  |
| positive | 2(3.8) | 1(3.3) |  | 1(7.7) | 4(17.4) |  |
| **PTEN** |  |  | 0.273 |  |  | 1.000 |
| negative | 49(92.5) | 25(83.3) |  | 10(83.3) | 21(87.5) |  |
| positive | 4(7.5) | 5(6.7) |  | 2(16.7) | 3(12.5) |  |
| **AURKA** |  |  | 0.617 |  |  | 0.635 |
| negative | 33(62.3) | 17(56.7) |  | 6(50.0) | 10(41.7) |  |
| positive | 20(37.7) | 13(43.3) |  | 6(50.0) | 14(58.3) |  |
| **BRCA1** |  |  | 0.903 |  |  | 0.813 |
| negative | 40(75.5) | 23(76.7) |  | 7(58.3) | 13(54.2) |  |
| positive | 13(24.5) | 7(23.3) |  | 5(41.7) | 11(45.8) |  |
| **BRCA2** |  |  | 0.738 |  |  | 0.378 |
| negative | 45(84.9) | 27(90.0) |  | 9(75.0) | 21(87.5) |  |
| positive | 8(15.1) | 3(10.0) |  | 3(25.0) | 3(12.5) |  |
| **PD-L1** |  |  | 0.477 |  |  | 0.114 |
| negative | 46(86.8) | 28(93.3) |  | 12(92.3) | 15(65.2) |  |
| positive | 7(13.2) | 2(6.7) |  | 1(7.7) | 8(34.8) |  |

There are 83 recurrent cases of HGSC and 36 recurrent cases of CCC having both primary tumor immunohistochemistry results and information of chemotherapy. However, the total number for each antibody does not add to up 83 and 36, respectively.

Chi-squared tests

Bold value denotes P with statistical significance.

**Table S4** Comparison of molecular biomarkers between recurrent and disease-free patients

| **Molecular biomarkers** | **HGSC** | | | **CCC** | | |
| --- | --- | --- | --- | --- | --- | --- |
|  | **Recurrent(%)** | **Disease-Free(%)** | **HGSC**  **P** | **Recurrent (%)** | **Disease-Free(%)** | **CCC**  **P** |
| **EGFR** |  |  | 0.072 |  |  | **0.023** |
| negative | 58(69.0) | 21(87.5) |  | 29（78.4） | 28(54.9) |  |
| positive | 26(31.0) | 3(12.5) |  | 8(21.6) | 23(45.1) |  |
| **HER2** |  |  | 1.000 |  |  | 0.929 |
| negative | 81（96.4） | 24（100.0） |  | 34（87.2） | 45（86.5） |  |
| positive | 3（3.6） | 0（0.0） |  | 5（12.8） | 7（13.5） |  |
| **PTEN** |  |  | 0.298 |  |  | 0.095 |
| negative | 75（89.3） | 19（79.2） |  | 32（82.1） | 48（94.1） |  |
| positive | 9（10.7） | 5（20.8） |  | 7（17.9） | 3（5.9） |  |
| **AURKA** |  |  | 0.596 |  |  | 0.265 |
| negative | 51（60.7） | 16（66.7） |  | 21（53.8） | 34（65.4） |  |
| positive | 33（39.3） | 8（33.3） |  | 18（46.2） | 18（34.6） |  |
| **BRCA1** |  |  | 0.182 |  |  | 0.969 |
| negative | 64（76.2） | 15（62.5） |  | 22（56.4） | 28（56.0） |  |
| positive | 20（23.8） | 9（37.5） |  | 17（43.6） | 22（44.0） |  |
| **BRCA2** |  |  | 0.293 |  |  | 0.731 |
| negative | 73（86.9） | 23（95.8） |  | 31(79.5) | 42(82.4) |  |
| positive | 11（13.1） | 1（4.2） |  | 8(20.5) | 9(17.6) |  |
| **PD-L1** |  |  | 0.718 |  |  | 0.224 |
| negative | 75（89.3） | 20（87.0） |  | 29(92.0) | 44(84.6) |  |
| positive | 9（10.7） | 3（13.0） |  | 10(8.0) | 8(15.4) |  |

There are 108 cases of HGSC and 91 cases of CCC having both primary tumor immunohistochemistry results (at least for 1 antibody) and information of recurrence. However, the total number for each antibody does not add to up 108 and 91, respectively.

Chi-squared tests

Bold value denotes P with statistical significance.
